# Study protocol: group-based psychoeducation for relatives of patients with bipolar disorder—a large scale real-world randomized controlled parallel group trial, the R-bipolar RCT

**DOI:** 10.1186/s13063-024-08172-z

**Published:** 2024-05-23

**Authors:** Julie Ravneberg Stokholm, Maj Vinberg, Maria Faurholt-Jepsen, Lars Vedel Kessing

**Affiliations:** 1 Psychiatric Center Copenhagen, The Copenhagen Affective Disorder Research Center (CADIC), Copenhagen, Denmark; 2 Department of Clinical Medicine, Faculty of Health and Medical Sciences, University of Copenhagen, Copenhagen, Denmark; 3 The Early Multimodular Prevention and Intervention Research Institution (EMPIRI), Mental Health Centre Northern Zealand, Hillerød, Denmark

**Keywords:** Bipolar and related disorders, Caregivers, Caregiver burden, Psychosocial intervention, Family

## Abstract

**Background:**

Relatives of patients with bipolar disorder (BD) often experience emotional burden with stress and depressive symptoms that again increase the likelihood of destabilization and relapses in the patient. The effects of group-based psychoeducation have not been investigated in large-scale real-world settings. We are currently conducting a large-scale real-world randomized controlled parallel group trial (RCT) to test whether group-based psychoeducation for 200 relatives to patients with BD improves mood instability and other critical outcomes in relatives and the corresponding patients with BD.

**Methods:**

The trial is designed as a two-arm, parallel-group randomized trial with a balanced randomization 1:1 to either group-based psychoeducation or a waiting list for approximately 4 months and subsequent group-based psychoeducation. The primary outcome measure is mood instability calculated based on daily smartphone-based mood self-assessments. Other relevant outcomes are measured, including patients’ reported outcomes, assessing self-assessed burden, self-efficacy, and knowledge about BD.

**Discussion:**

This protocol describes our currently ongoing randomized controlled trial (RCT) that aims at investigating group-based psychoeducation as an intervention for relatives of individuals diagnosed with bipolar disorder (BD). The study is the first large-scale real-world RCT to focus on a relatively short intervention of psychoeducation (6 sessions of 2 h each) in a large group of relatives (approximately 30 participants per group). With this focus, we wish to test an intervention that is feasible to implement in real-life psychiatric settings with limited budgets and time. It is also the first study to use mood instability in relatives as the primary outcome measure and to investigate whether mood instability and other affective symptoms in patients and relatives covary. It could be considered as limitations, that the trial is not blinded and does not include long-term follow-up.

**Trial registration:**

ClinicalTrials.gov NCT06176001. Registered on 2023–12-19. The study is approved by the data agency (P-2021–809). The project was allowed to be initiated without permission from the Scientific Ethical Committees for the Capital Region, because it according to section 1, paragraph 4 of the Committee Act was not defined as a health scientific intervention study (case number 21063013).

**Supplementary Information:**

The online version contains supplementary material available at 10.1186/s13063-024-08172-z.

## Administrative information

Note: the numbers in curly brackets in this protocol refer to SPIRIT checklist item numbers. The order of the items has been modified to group similar items (see http://www.equator-network.org/reporting-guidelines/spirit-2013-statement-defining-standard-protocol-items-for-clinical-trials/).

**Table:** 

Title {1}	Study protocol: group-based psychoeducation for relatives of patients with bipolar disorder—a large scale real-world randomized controlled parallel group trial, the R-bipolar RCT
Trial registration {2a and 2b}	ClinicalTrials.gov identifier: NCT06176001 For the World Health Organization Trial Registration Data Set see appendix 1
Protocol version {3}	23.11.2023, version 1
Funding {4}	The project is funded by the Research Fund of Mental Health Services—Capital Region of Denmark.
Author details {5a}	Julie Ravneberg Stokholm M.D.^1,2^ Maj Vinberg, MD, Professor, PhD., DMSc.^2,3^ Maria Faurholt-Jepsen, Associate professor, MD, DMSc.^1,2^ Lars Vedel Kessing, Professor, MD, DMSc. ^1,2^ ^1^ Psychiatric Center Copenhagen, The Copenhagen Affective Disorder Research Center (CADIC), Denmark ^2^ Department of Clinical Medicine, Faculty of Health and Medical Sciences, University of Copenhagen ^3^ The Early Multimodular Prevention and Intervention Research Institution (EMPIRI), Mental Health Centre Northern Zealand
Name and contact information for the trial sponsor {5b}	Lars Vedel Kessing, Professor, MD, DMSc. Psychiatric Center Copenhagen, The Copenhagen Affective Disorder Research Center (CADIC) Hovedvejen 17, 1. Sal, 2000 Frederiksberg, Denmark Tel: 0045 38 64 70 81 e-mail: lars.vedel.kessing@regionh.dk
Role of sponsor {5c}	The funding source had no role in the design of the study, its execution, analysis, interpretation of the data, or submission.

## Introduction

### Background and rationale {6a}

#### Bipolar disorder—impact on patients’ lives

Bipolar disorder (BD) is a common psychiatric disorder with a prevalence of 1–2% and a substantial heritability of 60–80% [1]. It ranks among the six leading causes of disability worldwide because it has an early age of onset and a life-long course characterized by a high risk of recurrence of manic and depressive episodes [2], a lifelong elevated risk of suicide, and a decreased life expectancy of 8–12 years [3, 4]. Current treatments do not sufficiently improve overall prognosis and approximately half of the patients experience long-term mood symptoms oscillating between depressive and hypomanic/manic symptoms [5] that severely impact functioning and quality of life [6, 7].

#### Bipolar disorder—impact on relatives

It is well-established that being a relative to a person with a severe mental disorder is burdensome with negative impacts on quality of life, finances, and mental health [8–13]. Specific for BD is its episodic nature and diverse symptoms in depressive, euthymic, hypomanic, manic, and mixed episodes, respectively, which contribute to the burden for relatives [14]. Even when the patient is euthymic, relatives still experience distress related to the patient’s problem behavior, e.g., hyperactivity, irritability, and withdrawal [15, 16].

As BD has shown high levels of heritability [1], family members who are genetically associated with the person with BD, might carry a genetic predisposition for psychiatric morbidity. Partners to patients with BD also have a 3 to 8 times increased risk of psychiatric disorders, which is thought to be partly due to assortative mating [17].

#### The prognosis of bipolar disorder is affected by relatives

Relatives provide vital emotional and practical support for patients and are important partners in the collaboration around treatment [18, 19]. Indeed, lower levels of social support in patients with BD have been shown to increase the risk of recurrence of depression [20] and the risk of only reaching partial recovery [21]. The emotional environment in the family also affects the patient’s prognosis and high levels of “expressed emotions” (criticism, hostility, and emotional overinvolvement) are prospectively associated with relapses in affective disorders [22, 23].

#### Group-based psychoeducation—effects on relatives/caregivers of patients with BD?

In relation to patients with BD, group-based psychoeducation is a first-line recommendation for the treatment of BD as an effective intervention as shown in 18 randomized controlled trials reducing illness recurrences, number and duration of hospitalizations, treatment adherence, therapeutic lithium levels, and reducing stigma [24, 25].

Several studies have been published on psychoeducational interventions for individual families [26–28], but offering psychoeducational interventions for individual families is expensive and poses high levels of organizational difficulties [29]. Group-based psychoeducation can reduce costs and organizational challenges, but as shown in the following overview, research on group-based psychoeducation exclusively for caregivers is scarce and often characterized by small study samples and/or small psychoeducation group sizes which again decreases feasibility.

The “Barcelona Bipolar Disorder Program psychoeducational intervention for caregivers of bipolar patients” has shown promising results in two RCTs from 2004 [30] to 2008 [31], respectively. The program involved 12 weekly sessions of 90 min in groups of approximately 10 caregivers. The results suggest improved knowledge of the illness and reduced subjective burden among 45 caregivers [30] and fewer recurrences among 113 patients who lived with their caregiver [31]. The program is however rather long and the groups small, which makes it both costly for the clinic to provide and time-consuming for the relatives to attend.

Subsequent studies have tested the effects of shorter programs. A study, with a rather small study sample of 47 relatives, compared the effects of three groups: a multifamily group psychoeducation (5 sessions), a solution-focused group therapy (5 sessions), and treatment as usual (TAU), and found increased knowledge and reduced burden in both intervention groups compared with TAU [32]. Another study from 2015 tested the efficacy of a psychoeducation program of 7 sessions (each 2 h), for caregivers in groups of 10 participants. The intervention was found to advance family members’ knowledge about the illness, alleviate their burden, and reduce their distress [33].

A small study with 32 participants from 2016 showed promising results with a reduction of caregivers’ burden and increased self-efficacy and knowledge after a very short intervention with 2 × 150 min of group-based psychoeducation for caregivers only [34]. A recent study from 2021 studied the effects of psychoeducation in 8 sessions in groups of 8–10 caregivers and showed effects on reduced burden on the relatives and increased function and decreased symptoms in the respective patients with BD [35]. A feasibility study from 2021 tested a 7-week program but had challenges in recruitment and therefore a very small study sample of just 12 participants [36].

A meta-analysis combining data from 9 studies covering individual, family, and group-based psychoeducation suggested that psychoeducation might improve caregiver burden but also that larger and more well-designed trials are needed before clinical recommendations can be made [37].

In this study, we aim to test the effect of a large-scale group-based psychoeducation intervention that has already been implemented in our clinic (see later) for ten years. It has shown to be feasible and affordable due to the relatively large groups of relatives of approx. 20–40 participants.

### Objectives {7}

#### Objectives

To conduct a pragmatic randomized controlled trial (RCT) investigating whether group-based psychoeducation for relatives to patients with BD improves mood stabilization and other critical outcomes, in relatives and in patients with BD.

#### Hypotheses

- *Primary:* group-based psychoeducation for relatives to patients with BD improves mood stabilization in *relatives*.
- *Secondary:* group-based psychoeducation for relatives to patients with BD improves other critical outcomes in *relatives* and mood stabilization and other critical outcomes in *patients with BD*.
- *Tertiary:* mood instability in patients and relatives covary.

### Trial design {8}

The trial is designed as a two-arm, parallel-group, superiority, randomized trial with a balanced randomization 1:1 to group-based psychoeducation versus a waiting list [38]. The control waiting list group will be offered participation in group-based psychoeducation (active intervention) during the following half year. The trial is planned and will be conducted in concordance with the CONSORT 2010 Explanation and Elaboration: updated guidelines for reporting parallel group randomized trials [39].

## Methods: participants, interventions, and outcomes

### Study setting {9}

The Copenhagen Affective Disorder Clinic is a large specialized mood disorder clinic with 18 full-time employees who provide treatment services for patients with newly diagnosed/first episode BD [40, 41]. The Copenhagen Affective Disorder Clinic receives patients from the entire Capital Region of Denmark covering a catchment area of 1.8 million people and all psychiatric centres in the region. Since 2010, Copenhagen Affective Disorder Clinic has provided large-scale group-based psychoeducation for relatives of patients with BD. Thus, we are investigating the effect of an intervention already implemented in real-world settings.

### Eligibility criteria {10}

- Inclusion criteria: relatives of patients with BD who are affiliated with the Copenhagen Affective Disorder Clinic. It is the patients who decide which relatives they would like to invite for psychoeducation.
- Exclusion criteria: Insufficient Danish language, or age under 18 years.

Data from *patients* with BD are collected from the A-bipolar trial, which is another RCT that is currently running in the Copenhagen Affective Disorder Clinic [42].

### Who will take informed consent? {26a}

JRS contacts the relatives by phone and informs them about the study. If they wish to participate, JRS sends written information about the R-bipolar study. At the following inclusion interview, an informed consent is signed by both the participant and the investigator conducting the interview. This investigator will primarily be JRS, but in case of high work-load or sickness absence, other members of the research group, who are trained in the relevant clinically rated observer-based scores (HAM, YMRS, and FAST) will perform the inclusions, including obtaining informed consent.

### Additional consent provisions for collection and use of participant data and biological specimens {26b}

N/A, we do not collect biological specimens.

## Interventions

### Explanation for the choice of comparators {6b}

We have chosen to compare the group-based psychoeducation intervention with a control group on a waiting list. We aim to investigate and evaluate clinical practice in an ongoing real-world intervention in comparison with its actual alternative: no group-based psychoeducation. The two-armed parallel group randomization also makes it possible to optimize the data yield because participants who are randomized to the control group will also participate in the intervention group afterwards.

### Intervention description {11a}

Group-based psychoeducation for relatives. Group size: 20–40 relatives. Duration of intervention: six sessions over 6–10 weeks, each session is 2 h long including a 15-min break. The sessions are held by experienced clinicians from the Copenhagen Affective Disorder Clinic, one chief physician, and one nurse. Each session focuses on a specific topic, which the clinicians present and discuss using a presentation viewer. The sessions are interactive, and the participants are encouraged to ask questions during the presentations. During each session, the participants will have some discussions in smaller groups to reflect on topics raised during the session.

The topics of the sessions are:

1. Introduction (Information about the clinic, introduction of the clinicians and participants, expectations alignments)
2. Bipolar disorder (the diagnosis and challenges in diagnosis)
3. Medical treatment (which medication is used and why)
4. Psychological treatment (psychoeducation for patients)
5. Living with a bipolar disorder (a guest lecturer with lived experience with BD tells his own story of living with BD)
6. Being a relative.

### Criteria for discontinuing or modifying allocated interventions {11b}

There are no criteria for discontinuing or modifying the allocated intervention for the individual participant.

### Strategies to improve adherence to interventions {11c}

The participating relatives are reminded about the research project because of their use of a smartphone-based monitoring system (The Monsenso app), which is presented below. After the psychoeducation, the participating relatives are asked to specify how many of the six sessions they attended, as a part of the final questionnaire.

### Relevant concomitant care permitted or prohibited during the trial {11d}

We cannot, nor should we, control if the participants seek guidance and information in other arenas.

### Provisions for post-trial care {30}

N/A. Owing to the nature of the intervention, we have no provisions.

### Outcomes {12}

The outcomes listed below are for (1) relatives to patients with BD and (2) the corresponding patients with BD.

**Fig. 1:**
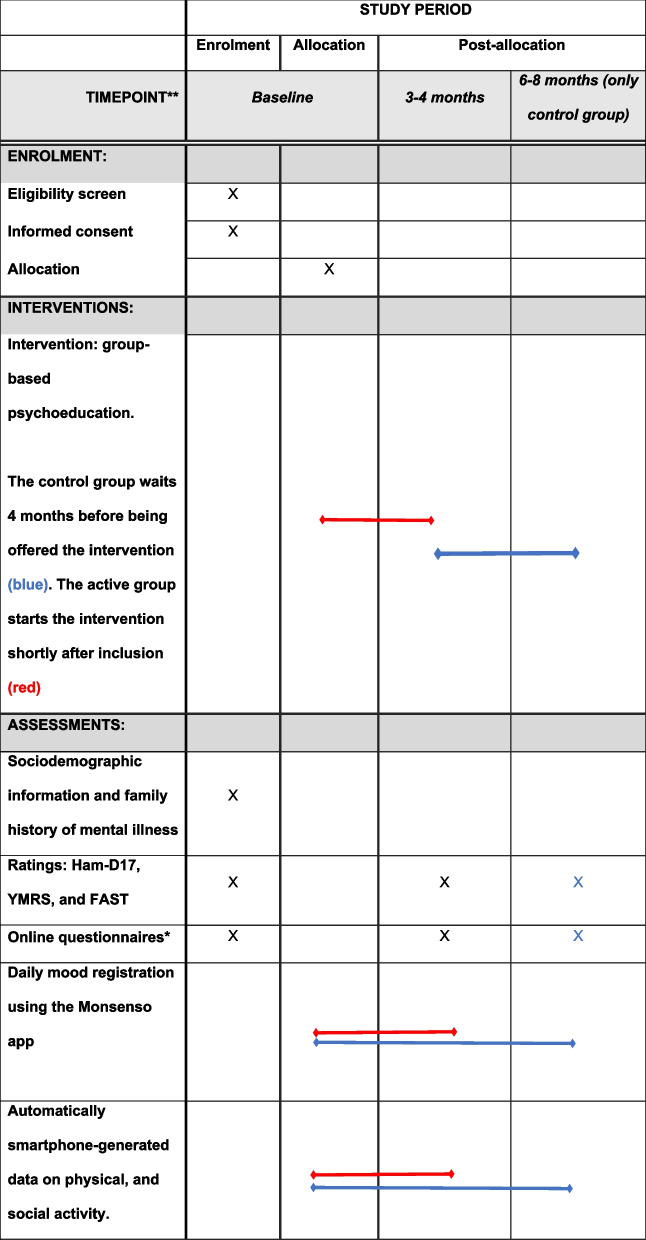
Spirit study schedule of enrolment, interventions, and assessments

Figure 1 shows an overview of the schedule of the study.

- *Primary outcome*: Daily self-reported mood instability collected via the Monsenso app^1^ [43] for relatives during the period in which the participants are part of the research project.
- *Secondary outcomes*: Other critically daily self-reported smartphone-based data including daily activity level, anxiety, irritability, stress, cognition, sleep, alcohol consumption, caregiver burden, and medicine as reported in [44].

Clinically rated observer-based scores on the following three scales: Hamilton Depression Scale-6 items (HAM-D6) [45] (being more sensitive in RCTs than the HAM-D17 [46]), the Young Mania Rating Scale (YMRS) [47] and the Functional Assessment Short Test (FAST) (a 24-item interviewer-administered interview concerning autonomy, occupational functioning, cognitive functioning, financial issues, interpersonal relationships and leisure time) [48].

Self-assessed scores on the following questionnaires: Burden Assessment Scale [49], Perceived Criticism Measure [22, 50], Carer Quality of Life [51, 52], Carer-Self-Efficacy [34, 53], Bipolar Knowledge Scale [54], Short Form-12 [55, 56], Brief Symptom Inventory [57], Perceived Stress Scale [58], Mood Disorder Questionnaire [59], Major Depression Inventory [60, 61], Childhood Trauma Questionnaire (CTQ) [62]. When the questionnaires are repeated after the intervention, the.

participants are asked to complete a satisfaction survey on the psychoeducation. The satisfaction survey was developed in close collaboration with the clinicians from the Copenhagen Affective Disorder Clinic.

- *Tertiary outcomes*: Automatically smartphone-generated data on physical [63], and social activity [64].

#### Smartphones as a new way to monitor treatment effects in research

We include smartphone-based self-assessment of mood instability as the primary outcome measure in the RCT for relatives. Similarly, we include data on mood instability for patients with BD. During the last ten years, we have developed and tested a unique smartphone-based system, the Monsenso system for monitoring, diagnosing, and treating BD. Digital electronic and remote self-monitoring of mood offers the possibility of ecological momentary assessments (EMA) [65, 66] for fine-grained real-time assessment in research settings. EMA reduces retrospective recall bias which is a particular problem for mood monitoring because patients need to recall both variation and intensity around a global mean [65]. Moreover, smartphone-based mood ratings in the Monsenso system correlate highly with clinical ratings of mood according to sub-item 1 on the Hamilton Depression Rating Scale 17-items (HAMD-17) and the Young Mania Rating Scale (YMRS), respectively (*p*’s < 0.0001) [67].

The Monsenso system collects time series consisting of data representing daily self-monitoring of mood, activity, sleep, irritability, cognitive problems, alcohol consumption, stress, and medication self-administration. In the version for relatives, medication administration is not included, instead, they are asked to what degree they have supported the patient and if the patient’s BD has been a burden today. Patients and relatives are prompted every evening to fill in these data. The process.

takes 2 min and we have previously shown that adherence to reporting is over 93% during a 6-month trial period [68] and over 72% during a 9-month trial period [69]**.** Besides self-reported data, we will collect daily *automatically* generated smartphone sensor data on physical [37], and social activity [38] that we have shown reflect illness activity.

#### Mood instability—a sensitive primary outcome measure in randomized controlled trials

We have selected mood instability as the primary outcome measure in the RCT as this has several advantages: mood instability has internal validity as a real-life measure for patients and high external validity as it reflects patients’ illness severity and functioning. Extensive evidence shows that mood stability is of core pathogenetic significance in BD [67, 70–73]. Thus, a substantial proportion of patients with BD and relatives experience subsyndromal daily mood swings that are associated with increased perceived stress, decreased quality of life and functioning [67, 73], and increased risk of relapse and hospitalization for patients [70–72]. Increased mood instability behaves as a genetic vulnerability trait for BD as it is present in remitted patients [73] and their unaffected relatives [67]. Accordingly, during the last decade, there has been a gradual shift from a focus on affective episodes to inter-episodic mood instability [74, 75]. Mood instability is currently considered a new treatment target in BD as it appears to be a more sensitive measure of outcome in RCTs than more conventional outcomes focusing on relapse or recurrence of affective episodes [74–76].

### Participant timeline {13}

The participant timeline is shown in Fig. 1.

### Sample size {14}

According to prior analyses [73] mood instability in patients with BD varies on a scale from close to 0 to 10 with an average of 4.1 (SD = 2.6). Our power calculation is based on the assumption that group-based psychoeducation will be associated with a minimum decrease of 0.2 in mood instability compared to the placebo arm; for a power of 80% and a type 1 error risk of 0.05, we need to randomize a total of 126 patients (www.sample-size.net). Also, we need to adjust for situations where one patient has two involved relatives, making them interdependent (estimated to be 30%), and consider expected dropout (estimated to be 10%, as in our prior study [68]) resulting in a total sample size of 180. We expect 200 relatives to agree in participating in the RCT.

### Recruitment {15}

Patients at Copenhagen Affective Disorder Clinical are routinely informed about the possibility of psychoeducation for their relatives. If interested, the patients will hand in a registration form with the name and phone numbers of their relative(s). The clinic generally recommends that patients start in psychoeducational group therapy themselves before their relatives are enrolled in psychoeducation. This is however only a recommendation and not a rule. Relatives’ psychoeducation groups are offered once in the autumn and two during spring.

Data from patients with BD are collected from the A-bipolar study, which is a parallel ongoing study [42]. We will secure written consent from the participants in the A-bipolar study to utilize their data in the R-bipolar study.

## Assignment of interventions: allocation

### Sequence generation {16a}

On the webpage www.randomization.com, we produced a randomization plan. The random numbers allocated the participants in either intervention (1) or control group (2).

We chose to have block randomization to achieve equal sizes of the groups during the ongoing recruitment process. If two participants were related to the same patient, they would be grouped together, as they would not be independent participants.

### Concealment mechanism {16b}

The randomization plan was downloaded as an Excel File, converted to a CSV file, and then uploaded to RedCap (https://www.project-redcap.org/). In RedCap a randomization module was set up, using the uploaded randomization plan.

### Implementation {16c}

JRS and MFJ generated the randomization plan. JRS enrolled the participants and assigned participants to intervention or control groups at the end of each inclusion visit.

## Assignment of interventions: blinding

### Who will be blinded {17a}

The study participants are not blinded. JRS is not blinded, but JRS attempts to minimize the potential influence by (a) assigning participants at the end of the inclusion visit, so that questionnaires and ratings at T0 are before the allocation. (b) JRS did not routinely check the participants’ group during the intervention.

### Procedure for unblinding if needed {17b}

Statistical analyses will be carried out blinded for randomization status (intervention versus control) until the results are clearly described.

## Data collection and management

### Plans for assessment and collection of outcomes {18a}

JRS will assess all participating relatives, unless sickness or an extraordinary busy period, requires assistance from a colleague. JRS has prior experience with the Hamilton rating and was trained in FAST and YMRS before the start of the trial. The questionnaires used are described in the outcome section {12}.

### Plans to promote participant retention and complete follow-up {18b}

We check weekly to see if the participants remember to record in the Monsenso App. If there is a lack of adherence, we contact participants by phone call or text message, whichever is most suitable in the case. JRS sends emails with a reminder and a link to the questionnaire in Redcap 1 to 2 weeks prior to follow-up interviews, thus increasing adherence. If a participant wishes to leave the study, the date and reason are noted in the primary logbook.

### Data management {19}

The researcher, primarily JRS, collects sociodemographic data, clinical data, and outcome measures (besides the primary) including questionnaires electronically and easily via the RedCap database. The RedCap database is a secure web application for building and managing databases and online surveys and is approved for use in research projects by the Capital Region of Denmark. A logbook with contact information, information on related patients, participant ID number, and inclusion status is kept in an Excel file on a logged drive. Also, a logbook used to check participants’ registration activity in the Monsenso app is kept in an Excel file on a logged drive. The researcher enters data directly into RedCap and the Logbook. Only registration forms and completed consent forms are handled in paper form and these are stored in a locked file cabinet.

When participants use the Monsenso app, the data is automatically available to the researchers on the Monsenso webpage (www.portal.monsenso.com), which requires a username and password. When the trial is completed, the researchers can retrieve data for the entire cohort of participants.

### Confidentiality {27}

At inclusion, all participants are given an individual identification number, which is used to link information between the database in RedCap, the logbook, and Monsenso. Only researchers associated with the R-bipolar project are given access to the project in RedCap. The logbook is kept on a logged drive to which only research members of the R-Bipolar study in the Copenhagen Affective Disorder research Centre (CADIC) have access.

### Plans for collection, laboratory evaluation, and storage of biological specimens for genetic or molecular analysis in this trial/future use {33}

N/A, biological specimens are not collected in this trial.

## Statistical methods

### Statistical methods for primary and secondary outcomes {20a}

#### Statistical analyses

Analyses will be conducted for the total sample of 200 relatives and their corresponding patients with BD. Two-level linear mixed-effect models will be used including a fixed effect of group (active group versus control) and a person-specific random effect allowing for individual variation in mood instability or other outcome measures.

In relation to hypothesis 3, it will be investigated whether mood instability in patients and relatives covary more than mood instability in patients and healthy control individuals (controls collected as part of the ongoing Bipolar Illness Onset (BIO) study in the Copenhagen Affective Disorder Clinic [77].

#### Estimation of mood instability

Estimates of instability will be based on readings obtained via the Monsenso system. Relatives and patients score their daily mood on a 9-point scale (patients on a scale from − 3 to + 3 and relatives on a scale 0–8). For each participant, a mood instability measure will be estimated for each day and aggregated in accordance with prior definitions by applying the root mean square successive difference (rMSSD) method [78–80]. The daily mood instability measures, reflecting the extent to which a daily and the previous day’s scores of self-monitored mood differ from one another during follow-up, will be computed as the squared successive difference (SSD) of the reported values. Consequently, daily instability measures can only be computed when consecutive daily values are present. The resulting SSD values are aggregated for each participant as the root mean square successive difference (rMSSD), taking the square root of the mean of the SSD values [80–83]. The instability measures will be computed following the original definition of the rMSSD score. The differences are squared such that positive and negative differences do not cancel out when the values are aggregated by computing the mean. Squaring the values also puts more weight on larger differences. Finally, the square root of the mean puts the aggregated value back on the original scale.

### Interim analyses {21b}

There is no planned interim analysis.

### Methods for additional analyses (e.g., subgroup analyses) {20b}

We plan to conduct an interaction analysis to test the effect of the type of relative (e.g., partner or parent) as well as cohabitation status between patient and relative.

### Methods in analysis to handle protocol non-adherence and any statistical methods to handle missing data {20c}

Missing data will be handled as missing-at-random, and therefore we will not conduct imputations strategies. Non-adherence will be minimized by checking adherence to smartphone-based monitoring continuously.

### Plans to give access to the full protocol, participant-level data, and statistical code {31c}

The datasets analyzed during the current study and statistical code will be made available from the corresponding author on reasonable request after publications from the R-Bipolar study, as is the full protocol.

## Oversight and monitoring

### Composition of the coordinating center and trial steering committee {5d}

Lars Vedel Kessing, Professor, MD, DMSc is the trial sponsor and principal investigator (see affiliation and contact information on the front page). The researcher JRS conducts the study in accordance with the protocol. MV and MFJ helped in developing the protocol and participated in the group that oversees the trial on an ongoing basis. Further, a total of ten relatives of the patients with bipolar disorder were involved and interviewed as part of a qualitative study.

### Composition of the data monitoring committee, its role and reporting structure {21a}

N/A, As this is not a clinical trial of medication, a data monitoring committee is not needed.

### Adverse event reporting and harms {22}

Although psychoeducation is not intended as therapy, there are therapeutic elements and thus also the possibility of adverse events [84]. After the sessions, the participants are asked to complete an online questionnaire that includes evaluation questions, where they can report any negative experiences. Also, parallel to the RCT, JRS performed qualitative interviews with ten participants to get a rich insight into the participants’ experiences with psychoeducation. The results from the qualitative analysis will be published in an independent paper.

### Frequency and plans for auditing trial conduct {23}

The research team including JRS, LVK, MV, and MFJ met every third month to review trial conduct that was discussed every 6 months with a greater research group consisting of 10–12 members.

### Plans for communicating important protocol amendments to relevant parties (e.g., trial participants, ethical committees) {25}

If substantive protocol amendments are needed, this will be discussed in the research group described in section {5d}: Composition of the coordinating center and trial steering committee {5d}. The principal investigator Lars Vedel Kessing will be responsible for the final decision, and amendments will be registered at the registration site and in the protocol.

### Dissemination plans {31a}

The findings will be widely disseminated at international conferences and meetings including conferences for the International Society for Bipolar Disorders (ISBD) and in scientific peer-reviewed papers. The research group also plans to host a meeting for all the relatives who have participated to present and discuss the results of the trial.

## Discussion

This protocol describes our currently ongoing randomized controlled trial (RCT) that aims at investigating group-based psychoeducation as an intervention for relatives of individuals diagnosed with bipolar disorder (BD). The study is the first large-scale real-world RCT to focus on a relatively short intervention of psychoeducation (6 sessions of 2 h each) in a large group of relatives (approximately 30 participants per group). With this focus, we wish to test an intervention that is feasible to implement in real-life psychiatric settings with limited budgets and time. It is also the first study to use mood instability in relatives as the primary outcome measure and to investigate whether mood instability and other affective symptoms in patients and relatives covary. It could be considered as limitations, that the trial is not blinded and does not include long-term follow-up.

## Trial status

Recruitment began on April 7th, 2022 and so far (January 5th, 2024) a total of 185 relatives have been included in the trial and 25 have dropped out. Recruitment is expected to be completed at the end of January 2024 and final data gathering will be finished in May 2024.

Protocol version 1.

### Supplementary Information

Supplementary material 1.

## Data Availability

Any data required to support the protocol can be supplied on request.

## Footnotes

1 Information about the Monsenso app can be found on the homepage: www.monsenso.com and the fact sheet: https://www.monsenso.com/wp-content/uploads/2023/05/Monsenso-Product-Overview.pdf
