# Supplementary material for: Study protocol: group-based psychoeducation for relatives of patients with bipolar disorder—a large scale real-world randomized controlled parallel group trial, the R-bipolar RCT
Source: Trials. 2024 May 23;25:342. doi: 10.1186/s13063-024-08172-z (PMC11119791; doi:10.1186/s13063-024-08172-z)
Supplement: Supplementary file 1 — Supplementary material 1. [file 13063_2024_8172_MOESM1_ESM.pdf]

**Items from the World Health Organization Trial Registration Data set (version 1.3.1.)** *https://www.who.int/clinical-trials-registry-platform/network/who-data-set*	
**Data category**	**Information**
1. Primary Registry and Trial Identifying Number	ClinicalTrials.gov identifier: NCT06176001
2. Date of Registration in Primary Registry	December 18th 2023
3. Secondary Identifying Numbers	Data agency: (P-2021-809).<br>Scientific Ethical Committee case number 21063013
4. Source(s) of Monetary or Material Support	The Research Fund of Mental Health Services - Capital Region of Denmark
5. Primary Sponsor	Lars Vedel Kessing, Professor, MD, DMSc
6. Secondary Sponsor(s)	n/a
7. Contact for Public Queries	Julie Ravneberg Stokholm M.D.[1,2]<br>Psychiatric Center Copenhagen, The Copenhagen Affective Disorder Research Center (CADIC)<br>Hovedvejen 17, 1. Sal, 2000 Frederiksberg, Denmark<br>Mail: Julie.ravneberg.stokholm@regionh.dk<br>Phone: 004523318604
8. Contact for Scientific Queries	Lars Vedel Kessing, Professor, MD, DMSc.<br>Psychiatric Center Copenhagen, The Copenhagen Affective Disorder Research Center (CADIC)<br>Hovedvejen 17, 1. Sal, 2000 Frederiksberg, Denmark<br>Tel: 0045 38 64 70 81<br>e-mail: lars.vedel.kessing@regionh.dk
9. Public Title	The effect of group-based psychoeducation for relatives of patients with bipolar disorder, examined using a randomized controlled trial.
10. Scientific Title	Study Protocol: Group-based psychoeducation for Relatives of patients with bipolar disorder – a large scale real-world randomized controlled parallel group trial, the R-bipolar RCT
11. Countries of Recruitment	Denmark
12. Health Condition(s) or Problem(s) Studied	Bipolar Disorder (BD) and caregivers' burden
13. Intervention(s)	Group-based psychoeducation for relatives of persons with bipolar disorder.
14. Key Inclusion and Exclusion Criteria	Relatives of BD patients affiliated with the Copenhagen Affective Disorder Clinic. Inclusion criteria: Danish speaking and adult >18 years.
15. Study Type	Randomized controlled trial
16. Date of First Enrollment	April 4th 2022
17. Sample Size	Goal: 200, recruited so far 183
18. Recruitment Status	Recruiting
19. Primary Outcome(s)	Changes in relatives' mood stability, self-reported through smartphone-app on a daily basis.
20. Key Secondary Outcomes	Other daily-reported smartphone-based data including: daily activity level, anxiety, irritability, stress, cognition, sleep, alcohol consumption, caregiver-burden, and medicine.<br>At inclusion and after intervention (approx. 7-8 months), if in control group: also a follow-up interview befor intervention (approx. 4 months) the following scores and questionnaires are completed:<br>Clinically rated observer-based scores on the following three scales: Hamilton Depression Scale-6 items (HAM-D6) (44), the Young Mania

	Rating Scale (YMRS) (46) and the Functional Assessment Short Test (FAST).<br>Self-assessed scores on the following questionnaires: Burden Assessment Scale (48), Perceived Criticism Measure(22,49), Carer Quality of Life (50,51), Carer-Self-Efficacy (34,52), Bipolar Knowledge Scale (53), Short Form-12 (54,55), Brief Symptom Inventory (56), Perceived Stress Scale (57), Mood Disorder Questionnaire (58), Major Depression Inventory (59,60).
21. Ethics Review	The study is approved by the data agency (P-2021-809). The project was allowed to be initiated without permission from the Scientific Ethical Committees for the Capital Region, because it according to section 1, paragraph 4 of the Committee Act was not defined as a health scientific intervention study (case number 21063013).
22. Completion date	Expected June 2024
23. Summary Results	No results yet
24. IPD sharing statement	No
